# Outcomes of Infectious versus Sterile Perforated Corneal Ulcers after Therapeutic Penetrating Keratoplasty in the United States

**DOI:** 10.1155/2016/6284595

**Published:** 2016-12-13

**Authors:** Sloan W. Rush, Ryan B. Rush

**Affiliations:** ^1^Panhandle Eye Group, 7400 Fleming Ave., Amarillo, TX 79106, USA; ^2^Texas Tech University Health Sciences Center, 1400 S. Coulter, Amarillo, TX 79106, USA; ^3^Southwest Retina Specialists, 7411 Wallace Blvd., Amarillo, TX 79106, USA

## Abstract

*Purpose*. To compare the long-term outcomes of infectious versus sterile perforated corneal ulcers after therapeutic penetrating keratoplasty in the United States.* Methods*. The charts of 45 consecutive eyes that underwent primary therapeutic penetrating keratoplasty for a perforated corneal ulcer at a single center were retrospectively reviewed. The perforated ulcers were classified as infectious or sterile and the underlying demographics, clinical features, and 36-month outcomes were compared among the two groups.* Results. *Mean follow-up among subjects was 38.6 (±6.9) months. Patients presenting with sterile perforated ulcers were more likely to have a peripheral perforation location (*p* = 0.0333) and recurrence of the underlying disease condition (*p* = 0.0321), require adjunctive surgical measures in the immediate postoperative period (*p* < 0.0001), have reperforation after keratoplasty (*p* = 0.0079), have worse best corrected visual acuity (*p* = 0.0130), develop no light perception vision (*p* = 0.0053), and require enucleation/evisceration (*p* = 0.0252) when compared to the infectious perforated ulcer group.* Conclusions*. Sterile perforated corneal ulcers have a worse prognosis and may be more frequent than those caused by infectious disease in the United States compared to the developing world.

## 1. Introduction

Corneal blindness has been a global topic of interest in recent years [1, 2], and corneal infection remains a major cause of corneal blindness, especially in the developing world [3, 4]. With improved economic development and access to care, countries in the developing world are becoming better equipped to treat and cure infectious corneal ulcers [5]. Nevertheless, in many cases, advanced infectious corneal ulcers may progress to corneal perforation [6], resulting in severe ocular morbidities and even loss of globe [7].

Therapeutic penetrating keratoplasty (TPK) remains the most vital treatment strategy for perforated infectious corneal ulcers [8–11]. Successful outcomes after TPK for perforated infectious corneal ulcers have been reported in the literature derived from the developing world [12]. However, in developed countries such as the United States where patient's access to healthcare resources and fortified antibiotics is greater, corneal ulcers apparently have a considerably lower incidence of perforation than in the developing world as evidenced by the sparse clinical data from the developed world [13, 14]. Furthermore, sterile corneal ulcers continue to be an important cause of corneal perforation in the United States and other developed nations [15, 16]. In this study, we compare the clinical course and long-term outcomes of infectious versus sterile perforated corneal ulcers after primary TPK in the United States.

## 2. Methods

The SRS Institutional Review Board (IORG0007600/IRB00009122) approved this retrospective, consecutive chart review that included all patients from August 2010 through August 2015 that received TPK for a perforated corneal ulcer at a single center in Amarillo, TX. All research components adhered to the tenets of the Declaration of Helsinki and were carried out in accordance with accepted human research regulations and standards.

### 2.1. Inclusion/Exclusion Criteria and Data Collection

The operative eyes of all patients that underwent primary TPK for a perforated corneal ulcer by a single surgeon (SWR) during the aforementioned study interval were included. Patients without completion of at least 18 months of follow-up and patients that underwent corneal gluing prior to TPK were excluded from the analysis. The baseline demographic features and characteristics, preoperative diagnoses with existing ocular comorbidities, and postoperative outcomes were collected. The baseline characteristics included subject age, gender, ethnicity, laterality, lens status, history of contact lens wear, location of perforation, and preoperative best corrected visual acuity (BCVA). The postoperative outcomes were collected over a 36-month follow-up period and included whether or not there was eradication of the underlying disease with initial TPK, the use and type of any adjunctive surgical measures to facilitate postoperative graft healing, the presence of graft clarity after initial TPK, whether or not reperforation occurred following the initial TPK, occurrence of regrafting after the initial TPK, the total number of corneal grafts received during the study interval, whether or not the patient received a Boston Type 1 Keratoprosthesis, the postoperative BCVA at 36 months, and whether or not the operative eye underwent enucleation/evisceration.

### 2.2. Perforated Corneal Ulcer Classification Criteria

The perforated ulcer was classified as central if the perforation was confined in its entirety within a 6 mm radius from the central apex of the cornea. The perforated ulcer was classified as peripheral if any portion of the perforation was located beyond a 6 mm radius from the central apex of the cornea. For perforated ulcers to be classified as infectious, the underlying pathogen must have been positively identified either by culture or else seen in the corneal button on pathology slides. Perforated ulcers classified as sterile all had negative culture results, clinical appearance without infiltrates or other findings that were suspicious for infectious agents, a clinically identifiable entity and known underlying pathology responsible for the ocular surface disease and corneal melting process that led to perforation, and corneal buttons that were negative for microorganisms when examined in the pathology lab. Various clinical findings including historical information, medications history, and corneal sensitivity testing were used to classify the underlying disease condition for the sterile perforated ulcers, but there was no consistent testing or method done uniformly for all of these patients.

### 2.3. Surgical Timing and Techniques

All TPK procedures were performed within 48 hours from the time in which the corneal perforation was diagnosed. A sixteen interrupted 10-0 nylon sutures' technique was used on all TPK surgeries. Graft size varied in diameter based upon the patient's existing anatomy and the location of the perforation but ranged between 7.5 and 10.0 mm. All donor corneal tissues had endothelial cell counts greater than 2,000 cells/mm^2^ according to preliminary eye bank testing. Adjunctive surgical measures done at the time of the TPK were discretionary according to the surgeon and sometimes included suturing or gluing of an amniotic membrane graft over the ocular surface and lateral suture tarsorrhaphy.

### 2.4. Statistical Analysis

The JMP 11 mathematical software package from the SAS Institute (Cary, NC, USA) was used to execute the statistical analysis and calculate means with standard deviations. The outcome variables were not assumed to have a normal distribution, so one-way analysis of the variance (and likelihood ratios, when appropriate for nominal variables) was used to compare the baseline characteristics and postoperative outcomes among the infectious and sterile perforated ulcer groups. Results were considered statistically significant at the alpha <0.05 level.

## 3. Results

A total of 45 eyes of 45 patients were included in the analysis. The mean age of the overall study population was of 58.2 (±21.1) years with 60% male. The mean follow-up among both groups collectively was 38.6 (±6.9) months (including 9 patients that deceased during the study interval).  Table 1 classifies all corneal ulcers as sterile and infectious and details the underlying diagnosis and pathology that led to corneal perforation requiring TPK. For the thirteen bacterial perforated ulcers, four cultures were positive for* Staphylococcus aureus* (two of them methicillin-resistant), two cultures were positive for* Haemophilus influenzae*, two cultures were positive for* Streptococcus pneumoniae*, two cultures were positive for* Pseudomonas aeruginosa*, and one culture each was positive for* Citrobacter koseri*,* Arthrobacter* spp., and a nonidentified atypical acid fast bacillus. For the seven fungal perforated ulcers, one culture each was positive for* Fusarium* spp.,* Cladorhinum* spp.,* Aspergillus* spp., and* Bipolaris* spp., and there were hyphae or other fungal elements identified on pathology specimen without positive culture for the remaining three cases.

Table 2 compares the baseline features and preoperative characteristics among the sterile and infectious cohorts. The infectious group was more likely to have a history of contact lens wear (*p* = 0.003) and previous keratoplasty (*p* = 0.0138), while the sterile group was more likely to have a peripheral location of the perforation site (*p* = 0.0333).  Table 3 details the postoperative outcomes of the two groups. Of note, patients presenting with sterile perforated ulcers were more likely to have recurrence of the underlying disease condition (*p* = 0.0321), have corneal reperforation (*p* = 0.0079), achieve worse BCVA (*p* = 0.0130), develop NLP vision (*p* = 0.0053), and eventually require enucleation/evisceration (*p* = 0.0252) when compared to the infectious perforated ulcer group during the study interval. The sterile ulcer group was also more likely than the infectious group to receive adjunctive surgical measures in the immediate postoperative period (*p* < 0.0001): 15 eyes received amniotic membrane grafting and 9 eyes received suture tarsorrhaphy in the sterile group, while just 1 eye received amniotic membrane grafting and 2 eyes received suture tarsorrhaphy in the infectious group. Subset analysis among the various sterile ulcer pathologies showed no significant difference in any of the different outcomes measured.

There were 9 study subjects (20%) who deceased during the 36-month study follow-up: three patients due to end stage cancer, two patients due to complications from chronic autoimmune disease, two patients due to end stage renal disease and other complications from diabetes, and two patients due to chronic heart disease. If the patient in either perforated ulcer group resided in a nursing home (*n* = 11), then they were more likely to decease (*p* = 0.0020) and develop worse BCVA (*p* = 0.0031) when compared the remainder of the study subjects. But nursing home residence did not correlate with either sterile or infectious perforated ulcer type (*p* = 0.1405). In addition, all phakic patients in both groups developed at least some degree of cataract progression during the follow-up interval, while three patients in the sterile group and two patients in the infectious group developed persistent increased intraocular pressure requiring topical medication.

## 4. Discussion

Primary TPK is considered the most definitive treatment option for large perforated corneal ulcers regardless of the underlying etiology [8–11], although recently there has been some interest in alternative techniques such as using autologous fibrin membrane combined with solid platelet-rich plasma [17], Tenons patch grafting [18], grafting with processed pericardium combined with synthetic materials [19], and partial thickness lamellar grafting techniques [20]. To our knowledge, this is the first case series to specifically compare long-term outcomes of primary TPK in the setting of sterile versus infectious perforated corneal ulcers in the developed world. Our data suggest that sterile corneal perforations may be more common than infectious corneal perforations in the United States. A recent study by Yokogawa et al. also reported a higher frequency of sterile perforated ulcers to infectious perforated ulcers in the developed world, but their study had too few cases in which primary TPK was performed to make a valid comparison to the results of our study [14]. Furthermore, we observed that the patient population most likely to develop a perforated corneal ulcer in the United States often has substantial baseline risk factors with an immunocompromised state such as end stage cancer, end stage renal disease, poorly controlled diabetes, an advanced autoimmune disorder, existing corneal graft, or residence in a nursing home. Our results indicate that not only is there severe ocular morbidity for many patients presenting with a perforated corneal ulcer in the United States, but also that there is increased mortality (20% of our study subjects) due to other preexisting systemic comorbidities that can be associated with the ocular disease. For these reasons, a valid comparison cannot be made among studies from the developing world where TPK is frequently performed prior to occurrence of corneal perforation for infectious ulcers that typically occur in otherwise healthy patients.

Amniotic membrane grafting, autologous serum topical therapy, and tarsorrhaphy have been used as adjunctive measures in the management of perforated corneal ulcers [21–23]. In our study, most of the sterile perforated ulcer patients received aggressive adjunctive measures in combination with TPK but still had worse visual and anatomic outcomes compared to the infectious perforated ulcer patients. This highlights the importance of earlier detection and treatment of sterile corneal ulceration to prevent more advanced disease and perforation from occurring. Nursing home residents with corneal ulcers in particular are an extremely vulnerable group requiring more prompt identification, attention, and specialized care before corneal perforation develops.

Peripheral ulcerative keratitis was the most common underlying cause of sterile ulcer perforation in this series. Patients with peripheral ulcerative keratitis often had other severe comorbidities due to their underlying rheumatologic disorder that likely contributed to delayed corneal healing after TPK [24]. In our series, three out of the seven patients that developed an autoimmune-related perforation were, at minimum, treated with 40 mg of daily oral prednisone at the presentation of their ulcer whereas the other four of these seven patients presented with large perforation already existing. All seven of the patients were continued on minimum of oral prednisone 40 mg daily for 2 weeks after TPK and tapered off over a period of weeks and sometimes months. During the same time period that this study was conducted, there were 5 other patients that had small perforated autoimmune-related ulcers that were treated with glue and never had TPK. Disease-modifying biologic agents may assist in the management in autoimmune disease that develops peripheral ulcerative keratitis, although further studies are needed to determine their impact on corneal ulcer perforation prevention [25].

Our study weaknesses include the retrospective data collection and the small number of study cases. In conclusion, patients in the United States undergoing TPK for a sterile perforated corneal ulcer are more likely to have the perforation in the peripheral cornea and recurrence of the underlying disease condition in the corneal graft, require adjunctive surgical measures in the immediate postoperative period, have reperforation after TPK, and have worse vision with loss of globe compared to patients undergoing TPK for an infectious perforated corneal ulcer. Future investigations are needed to further validate the findings reported in this study.

## Figures and Tables

**Table 1:** Underlying ulcer pathologies that led to corneal perforation requiring therapeutic penetrating keratoplasty.

Corneal ulcer type	Preoperative diagnosis
Sterile (*n* = 25)	Peripheral ulcerative keratitis due to an underlying autoimmune condition
	Associated with rheumatoid arthritis (*n* = 5)
	Associated with systemic lupus erythematosus (*n* = 2)
	Underlying neurotrophic keratopathy
	Associated with previous herpes zoster keratoconjunctivitis (*n* = 3)
	Associated with diabetic neuropathy (*n* = 2)
	Associated with nerve palsy after brain neoplasm excision (*n* = 1)
	Associated with dry eye syndrome and other chronic ocular surface disease (*n* = 4)
	Acquired limbal stem cell deficiency due to previous external beam
	Radiation to periocular skin neoplasm (*n* = 2)
	Graft versus host disease after bone marrow transplantation (*n* = 2)
	Stevens Johnson syndrome (*n* = 2)
	Toxicity of topical nonsteroidal anti-inflammatory drug (*n* = 1)
	Traumatic alkaline chemical injury (*n* = 1)

Infectious (*n* = 20)	Bacterial keratitis
	Associated with contact lens wear (*n* = 4)
	Occurring after previous corneal transplantation (*n* = 5)
	Associated with other chronic ocular surface diseases (*n* = 4)
	Fungal keratitis
	Associated with contact lens wear (*n* = 4)
	Occurring after previous corneal transplantation (*n* = 1)
	Associated with other chronic ocular surface diseases (*n* = 2)

**Table 2:** Comparison of baseline demographic features and preoperative characteristics of sterile and infectious perforated corneal ulcers that required therapeutic penetrating keratoplasty (with 95% confidence intervals).

Demographic features and preoperative characteristics	Sterile ulcers (*n* = 25)	Infectious ulcers (*n* = 20)	*p* value
Age (years)	57.7 (49.1–66.3)	58.8 (49.2–68.4)	0.8617
	Range = 19 to 86	Range = 18 to 91	

Gender	Male = 56% (*n* = 14)	Male = 65% (*n* = 13)	0.5394
	Female = 44% (*n* = 11)	Female = 35% (*n* = 7)	

Laterality	Right eye = 56% (*n* = 14)	Right eye = 45% (*n* = 9)	0.4629
	Left eye = 44% (*n* = 11)	Left eye = 55% (*n* = 11)	

Ethnicity	White = 60% (*n* = 15)	White = 85% (*n* = 17)	0.1108
	Hispanic = 28% (*n* = 7)	Hispanic = 5% (*n* = 1)	
	Black = 8% (*n* = 2)	Black = 10% (*n* = 2)	
	Asian = 4% (*n* = 1)	Asian = 0% (*n* = 0)	

Lens status	Phakic = 72% (*n* = 18)	Phakic = 60% (*n* = 12)	0.3968
	Pseudophakic = 28% (*n* = 7)	Pseudophakic = 40% (*n* = 8)	

Contact lens wear	Yes = 0 (0%)	Yes = 7 (35%)	0.0003
	No = 25 (100%)	No = 13 (65%)	

Previous corneal graft	Yes = 1 (4%)	Yes = 6 (30%)	0.0138
	No = 24 (96%)	No = 14 (70%)	

Location of perforation	Central = 72% (*n* = 18)	Central = 95% (*n* = 19)	0.0333
	Peripheral = 28% (*n* = 7)	Peripheral = 5% (*n* = 1)	

Preoperative best corrected visual acuity (logMAR)	2.64 (2.49–2.79)	2.65 (2.48–2.82)	0.9311

**Table 3:** Comparison of postoperative outcomes of sterile versus infectious perforated corneal ulcers that required therapeutic penetrating keratoplasty (with 95% confidence intervals).

Postoperative outcomes	Sterile ulcers (*n* = 25)	Infectious ulcers (*n* = 20)	*p* value
Eradication of underlying disease with initial therapeutic penetrating keratoplasty	Yes = 14 (56%) No = 11 (44%)	Yes = 17 (85%) No = 3 (15%)	0.0321

Adjunctive surgical measures required to facilitate postoperative graft healing	Yes = 19 (76%) No = 6 (24%)	Yes = 3 (15%) No = 17 (85%)	<0.0001

Clarity achieved on the first graft	Yes = 5 (20%) No = 20 (80%)	Yes = 7 (35%) No = 13 (65%)	0.2589

Reperforation occurred after initial therapeutic penetrating keratoplasty	Yes = 9 (36%) No = 16 (64%)	Yes = 1 (5%) No = 19 (95%)	0.0079

Regrafted again after initial therapeutic penetrating keratoplasty	Yes = 12 (48%) No = 13 (52%)	Yes = 8 (40%) No = 12 (60%)	0.5910

Total number of corneal grafts received	1.9 (1.4–2.5) Range = 1 to 8	1.7 (1.1–2.3) Range = 1 to 5	0.6068

Eventually received Boston Type 1 Keratoprosthesis	Yes = 6 (24%) No = 19 (76%)	Yes = 2 (10%) No = 18 (90%)	0.2112

Postoperative best corrected visual acuity (logMAR) at 3-year follow-up	2.43 (2.04–2.82) Range = 0.1 to 3.5	1.67 (1.21–2.11) Range = 0 to 3	0.0130

Operative eye became no light perception	Yes = 6 (24%) No = 19 (76%)	Yes = 0 (0%) No = 20 (100%)	0.0053

Operative eye received enucleation/evisceration	Yes = 4 (16%) No = 23 (84%)	Yes = 0 (0%) No = 20 (100%)	0.0252

Patient deceased during study interval	Yes = 6 (24%) No = 19 (76%)	Yes = 3 (15%) No = 17 (85%)	0.4487
